# Trophectoderm Transcriptome Analysis in LIN28 Knockdown Ovine Conceptuses Suggests Diverse Roles of the LIN28-let-7 Axis in Placental and Fetal Development

**DOI:** 10.3390/cells11071234

**Published:** 2022-04-05

**Authors:** Asghar Ali, Muhammad A. Iqbal, Muhammad W. Abbas, Gerrit J. Bouma, Russell V. Anthony, Thomas E. Spencer, Quinton A. Winger

**Affiliations:** 1 Animal Reproduction and Biotechnology Laboratory, Department of Biomedical Sciences, 1683 Campus Delivery, Colorado State University, Fort Collins, CO 80523, USA; gerrit.bouma@colostate.edu (G.J.B.); russ.anthony@colostate.edu (R.V.A.); 2 Chair for Molecular Animal Breeding and Biotechnology, Gene Centre, Ludwig Maximilian University of Munich (LMU), Feodor-Lynen-Strasse-25, 81377 Munich, Germany; 3 Institute of Genome Biology, Research Institute for Farm Animal Biology, 18196 Dummerstorf, Germany; iqbal@fbn-dummerstorf.de; 4 School of Interdisciplinary Engineering and Sciences, National University of Science and Technology, Islamabad 44000, Pakistan; abbas.waseem.gcu@gmail.com; 5 Animal Science Research Center, College of Agriculture, Food and Natural Resources, University of Missouri, Columbia, MO 65211, USA; spencerte@missouri.edu

**Keywords:** microRNAs, blastocyst, RNA sequencing, LIN28A, LIN28B, fetal growth

## Abstract

The proper conceptus elongation in ruminants is critical for the successful placentation and establishment of pregnancy. We have previously shown that the trophectoderm-specific knockdown of LIN28A/B in day 9 ovine blastocysts resulted in increased let-7 miRNAs and reduced conceptus elongation at day 16 of gestation. In this current study, by transcriptome analysis of LIN28A knockdown (AKD) or LIN28B knockdown (BKD) trophectoderm (TE), we explored the downstream target genes of the LIN28-let-7 axis and their roles in the placental and fetal development. We identified 449 differentially expressed genes (DEGs) in AKD TE and 1214 DEGs in BKD TE compared to non-targeting control (NTC). Our analysis further revealed that 210 downregulated genes in AKD TE and 562 downregulated genes in BKD TE were the potential targets of let-7 miRNAs. Moreover, 16 downregulated genes in AKD TE and 57 downregulated and 7 upregulated genes in BKD TE were transcription factors. The DEGs in AKD and BKD TE showed enrichment in the biological processes and pathways critical for placental development and function, and fetal development and growth. The results of this study suggest the potential roles of the LIN28-let-7 axis in placental and fetal development beyond its involvement in trophoblast proliferation and conceptus elongation.

## 1. Introduction

Embryonic mortality is one of the major factors affecting reproductive performance in mammals. In ruminants, 20–40% of pregnancies are lost before birth, most of which happen during the first three weeks of gestation [1,2]. The stages of conceptus development and establishment of pregnancy are similar in cattle and sheep. In sheep, the blastocyst hatches out of zona pellucida at day 8-9 post-conception and is covered by a single layer of mononucleated trophoblast cells called trophectoderm (TE) [3]. After zona hatching, the blastocyst develops into an ovoid or tubular conceptus comprising an embryo and the extraembryonic membranes [1]. From day 11–16 of gestation, the conceptus elongates primarily due to the rapid proliferation of trophoblast cells and becomes up to 25 cm long by day 16 [4,5,6]. Between days 14–16 of gestation, the binucleate trophoblast cells (BNCs) start appearing in TE and are developed by acytokinetic mitosis of the mononuclear trophoblast cells [7]. By days 16–18, the conceptus implantation starts with the adhesion of trophectoderm cells to the uterine epithelial cells in the maternal caruncles [8]. The BNCs fuse with the luminal epithelial cells to form the conceptus-maternal hybrid syncytial plaques which eventually form an epithelial interface between cotyledonary and caruncular tissues within the sheep placentome [9]. The proper conceptus elongation is essential for the successful implantation, placentation, and establishment of pregnancy [10], whereas the reduced conceptus length and impaired placentation are the major causes of embryonic mortality and reduced fertility in domestic animals [11,12,13].

The placenta is the only channel for exchanging nutrients and gases between the mother and fetus [14]. The major transplacental transport in ruminants occurs through placentomes, and the trophoblast cells in placentomes and interplacentomal regions differ in functionality and gene expression patterns [15]. Besides their role in the transplacental transport, the trophoblast cells also produce some important hormones, growth factors, and proteins [4,6]. Interferon-tau produced by the trophoblast cells plays an essential role in the pregnancy recognition in ruminants [16], and its peak production in sheep is at day 16 of gestation [17]. The BNCs in sheep TE secrete ovine placental lactogen (OPL) or chorionic somatomammotropin (CSH) in both fetal and maternal circulation [18], an important hormone that has been associated with fetal growth and metabolism in sheep [19,20].

In addition to regulating the trophectoderm function, the trophoblast-specific microRNAs (trophomiRs) and other families of microRNAs (miRNAs) produced in trophoblast cells are exported to both maternal and fetal circulation during pregnancy [21]. We recently reviewed the role of miRNAs in the pathogenesis of pregnancy complications, and their potential to disrupt important biological processes in both mother and fetus [22]. An important family of miRNAs with profound roles in trophoblast function is the lethal-7 (let-7) family of miRNAs [22,23,24,25]. Let-7 miRNAs, also known as differentiation-inducing miRNAs, are highly expressed in differentiated cells [26]. By binding the complimentary regions in mRNAs, let-7 miRNAs reduce the expression of a wide range of genes, including different proliferation factors [23,27]. The biogenesis of let-7 miRNAs is inhibited by the oncoprotein LIN28 [28]. LIN28 is a highly conserved RNA-binding protein that is highly expressed in undifferentiated cells and has two paralogs, LIN28A and LIN28B [29,30].

LIN28 knockout in the first-trimester human trophoblast cells leads to upregulation of let-7 miRNAs, downregulation of proliferation-associated genes, and reduced cell proliferation [23]. We have previously shown that the trophectoderm-specific knockdown of LIN28A or LIN28B in ovine blastocyst increases the expression of let-7 miRNAs and reduces the conceptus elongation [24]. In this study, we identified the differentially expressed genes (DEGs) in LIN28A/B knockdown TE by transcriptome analysis. We also performed the downstream functional analysis of the DEGs to explore the potential roles of the LIN28-let-7 axis in placental and fetal development beyond its involvement in conceptus elongation. We hypothesized that the LIN28-let-7 axis regulates the key biological processes during placental and fetal development by regulating the expression of its downstream target genes.

## 2. Materials and Methods

### 2.1. Trophectoderm Collection from Day 16 Conceptuses and RNA Sequencing

We have previously reported the detailed procedure for the generation of pregnancies with trophectoderm-specific LIN28A/B knockdown [24]. Briefly, day 9 hatched ovine blastocysts were incubated with lentivirus particles expressing a non-targeting shRNA or an shRNA to target either LIN28A or LIN28B mRNA. The shRNA sequences are provided in Table S1. After 4–5 hours of incubation with lentivirus particles at 5% CO_2_, 5% O_2_, and 38.5 °C, the blastocysts were transferred to the estrus synchronized recipients with 1 blastocyst per recipient using our previously described protocol [24,31]. Non-targeting control (NTC), LIN28A knockdown (AKD), and LIN28B knockdown (BKD) pregnancies were generated. Day 16 conceptuses were collected, embryos were separated, and the trophectoderm (TE) was used for total RNA extraction using an RNeasy mini kit with on-column DNAase treatment (Qiagen, Hilden, Germany).

The quality of the total RNA extracts from NTC, AKD, and BKD TE (n = 3 per group) was determined using RNA 6000 Pico kit and Bioanalyzer 2100 (Agilent Technologies, Santa Clara, CA, USA). For mRNA enrichment, the samples containing total RNA were subjected to selective depletion of ribosomal RNA using Ribo Minus Eukaryote kit (Thermo Fisher Scientific Inc., Hillsboro, OR, USA). The TruSeq stranded mRNA HT library construction procedure (Illumina) was used to prepare and sequence the libraries at the Genomic Technology Core Facility of the University of Missouri. The library was validated using the Fragment Analyzer (Advanced Analytical) with HiSens Next Generation Sequencing (NGS) reagents and quantitated using Qubit HS DNA assay kit (Thermo Fisher Scientific, Inc.) and quantitative PCR library kit (Kappa Biosystems, Inc., Wilmington, MA, USA). The libraries were pooled and sequenced on the HiSeq 2500 platform (Illumina) as 100-base pair (bp) paired-end sequences. 

### 2.2. Data Pre-Processing

Genomics Server 6.0 and Genomics Workbench 7.0.4 (CLC bio) were used to analyze RNA-Seq data. Using the default parameters, we quality-trimmed the Ion reads (error probability: 0.02) and Illumina reads (error probability: 0.001), and 13 bps were trimmed from the 5ʹ-end of each read. The genome assembly of Ovis aries (Oar_v3.1) was used for mapping the sequencing libraries using Ensembl annotations version 75, requiring paired mapping and using fragment per kilobase of transcript per million (FPKM) value as an expression metric. A box plot of square root-transformed expression values was used to assess the quality control, ensuring that all samples showed similar distributions. Scatter plots were generated in the R programming environment (version 4.0.3) to identify the differentially expressed genes (DEGs) in AKD and BKD TE compared to NTC. Only the genes with |log fold change (logFC) ≥ 2| and FPKM ≥ 5 were considered as the DEGs. The heatmap of RNA expression profiles in different samples was generated using hierarchical clustering by heatmap.2 function of gplots package (version 3.0.1) in the R programming environment. The heatmap facilitated a comparison of expression profiles of the DEGs and transcription factors between different experimental groups.

### 2.3. Identification of the Transcription Factors in the DEGs

The Animal Transcription Factor Database (AnimalTFDB; version 3.0) was used to obtain a list of Ovis aries transcription factors [32]. Venn plots were used to identify common genes between the Ovis aries transcription factors and the DEGs in AKD vs. NTC, BKD vs. NTC, and BKD vs. AKD comparisons.

### 2.4. Let-7 miRNAs and Prediction of Their Downstream Target Genes

The procedure and data of let-7 miRNAs quantification have been described in our previous study [24]. To find the target genes of let-7 miRNAs in the downregulated genes in AKD and BKD TE, 1027 coding, 692 3’-UTR, and 611 5’-UTR sequences were obtained from the Ovis aries genome (Oar_rambouillet_v1.0) using Ensembl annotation version 102. All obtained sequences were converted to 2000-bps fragments with an overlap of 50 bps. The target genes of let-7 miRNAs were identified using RNAhybrid version 2.1.2. The complete sequence of each miRNA was used for its target identification with parameters set as a single hit per target, minimum free energy (MFE) < −25 kcal/mole, helix constraint from base 2 to 7, and human-based assumed *p*-value distribution [33,34]. Only the list of downregulated DEGs was used to identify let-7 target genes, and the resultant pairs were used for further analyses. MetScape plugin (version 3.1.3) in the Cytoscape environment (version 3.8.2) was used to generate a network of the let-7 miRNAs and their target genes.

### 2.5. Gene Ontology and KEGG Pathway Enrichment Analysis

The DEGs in AKD and BKD TE were used for gene ontology (GO) enrichment analysis for biological processes (BPs) using DAVID bioinformatics resources (version 6.8) [35]. A visualization chart of the BPs related to placental and fetal development was generated using the goplot package (version.1.0.2) in the R programming environment (version 4.0.3). Cytoscape plugins, ClueGO (version 2.5.1) and Cluepedia (version 1.5.7) were used for KEGG pathway enrichment analysis of the DEGs [36,37,38]. KEGG pathways related to placental and fetal development were visualized as bar graphs generated using GraphPad Prism 9 software. To find enriched hallmark pathways and reactome pathways by the DEGs, the Gene Set Enrichment Analysis software (version 4.1.0) was used. A cut-off of *p* ≤ 0.05 was used to identify significantly enriched GO terms and KEGG pathways.

The common DEGs in all three possible comparisons (AKD vs. NTC, BKD vs. NTC, and BKD vs. AKD) were identified using Venn plots and clustered using heatmap.2 function of gPlots package (version 3.0.1) [39]. Similarly, differentially expressed transcription factors in AKD and BKD TE were identified by Venn plots and clustered using heatmap.2 function of gPlots package (version 3.0.1) [39]. The common DEGs clusters, transcription factors clusters, and the let-7 target genes were also subjected to KEGG pathway and GO enrichment analysis using ClueGO and Cluepedia Cytoscape plugins.

## 3. Results

### 3.1. Differentially Expressed Genes

**Figure 1:**
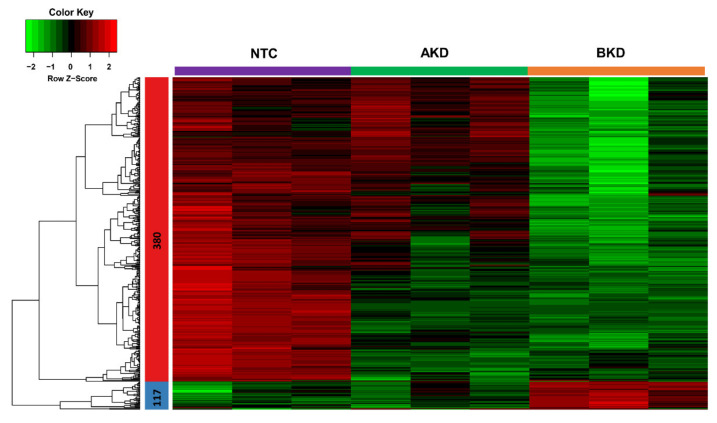
Heatmap of mRNAs expression profiles in non-targeting control (NTC), LIN28A knockdown (AKD), and LIN28B knockdown (BKD) trophectoderm (TE). Each column represents the expression profile of genes in a single day 16 TE. A total of 497 genes were distributed in two clusters based on their co-expression with cut-off criteria of FPKM ≥ 5, |logFC ≥ 5|. In the color key, the red color represents upregulation, the green color represents downregulation, and the black color represents no change in expression.

**Figure 2:**
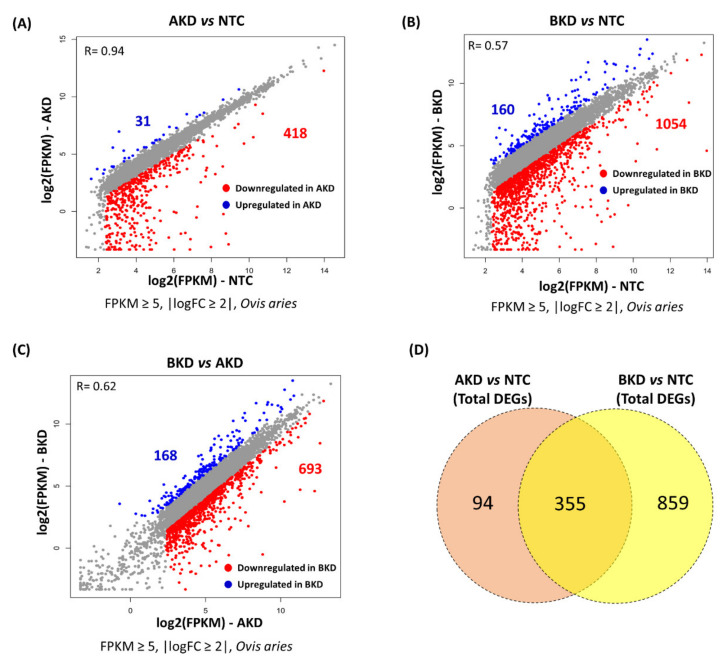
Scatter plots to identify the DEGs in: (**A**) AKD vs. NTC, (**B**) BKD vs. NTC, and **(C)** BKD vs. AKD, with cut-off criteria of FPKM ≥ 5, |logFC ≥ 2|. The red dots represent downregulated genes, the blue dots represent upregulated genes, and the grey dots represent the genes that did not show any change between different groups. (**D**) Venn plot comparing the total DEGs in AKD vs. NTC and BKD vs. NTC.

A heatmap was generated to compare the overall expression of genes in NTC, AKD, and BKD TE. A total of 497 genes were identified as the DEGs with cut-off criteria of FPKM ≥ 5, |logFC ≥ 5|, and were distributed in two clusters based on their co-expression, including 380 genes in cluster 1 and 117 genes in cluster 2 (Figure 1). The expression profile of genes in the heatmap showed that, compared to NTC, more genes were differentially expressed in BKD than AKD TE. The genes in cluster 1 were downregulated, and those in cluster 2 were upregulated in BKD compared to NTC TE. We further analyzed the differential expression of genes by scatter plots, and genes with FPKM ≥ 5 and |logFC ≥ 2| were considered differentially expressed. A total of 418 genes were downregulated and 31 genes were upregulated in AKD compared to NTC TE (Figure 2A), 1054 genes were downregulated and 160 genes were upregulated in BKD compared to NTC TE (Figure 2B), and 693 genes were downregulated and 168 genes were upregulated in BKD compared to AKD TE (Figure 2C, Tables S2–S4). A total of 355 genes were differentially expressed in both AKD and BKD compared to NTC TE. The DEGs obtained from the scatter plot analysis were used for downstream enrichment analysis.

### 3.2. Downstream Enrichment Analysis of the DEGs

**Figure 3:**
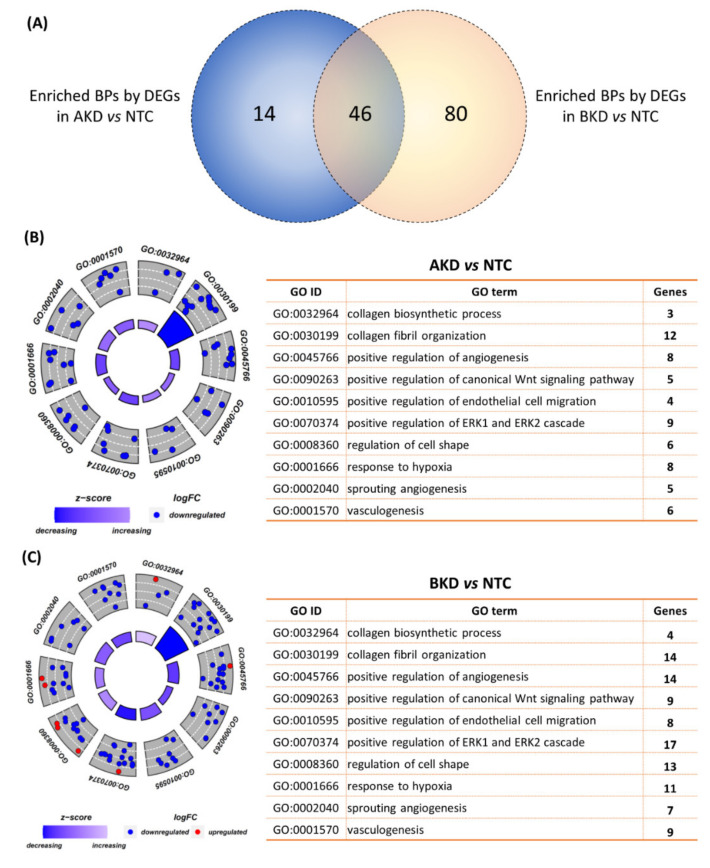
GO enrichment analysis for biological processes of the DEGs in AKD vs. NTC and BKD vs. NTC. (**A**) Venn plot of the BPs significantly enriched by the DEGs in AKD vs. NTC and BKD vs. NTC. The figure also shows 10 BPs related to placental or fetal growth significantly enriched by the DEGs in (**B**) AKD vs. NTC and (**C**) BKD vs. NTC. The blue dots indicate downregulated genes, the red dots indicate upregulated genes, and the white dotted line indicates the partition of logFC from low to high level. A complete list of BPs significantly enriched by the DEGs in AKD and BKD TE is provided in Tables S2 and S3.

The DEGs in AKD vs. NTC and BKD vs. NTC were separately subjected to GO enrichment analysis focusing on biological processes (BPs), and the BPs with *p* ≤ 0.05 were considered significantly enriched. The DEGs in AKD TE significantly enriched 60 BPs and the DEGs in BKD TE significantly enriched 126 BPs, whereas there were 46 common enriched BPs in both AKD and BKD TE (Figure 3A). Some of the BPs involved in placental development, enriched in both AKD and BKD TE, included collagen biosynthesis process, collagen fibril organization, positive regulation of angiogenesis, organization, positive regulation of canonical Wnt signaling pathway, positive regulation of endothelial cell migration, positive regulation of ERK1 and ERK2 cascade, regulation of cell shape, response to hypoxia, sprouting angiogenesis, and vasculogenesis (Figure 3B,C; Tables S2 and S3). These pathways were enriched mainly by downregulated genes in both AKD and BKD TE (Figure 3B,C).

**Figure 4:**
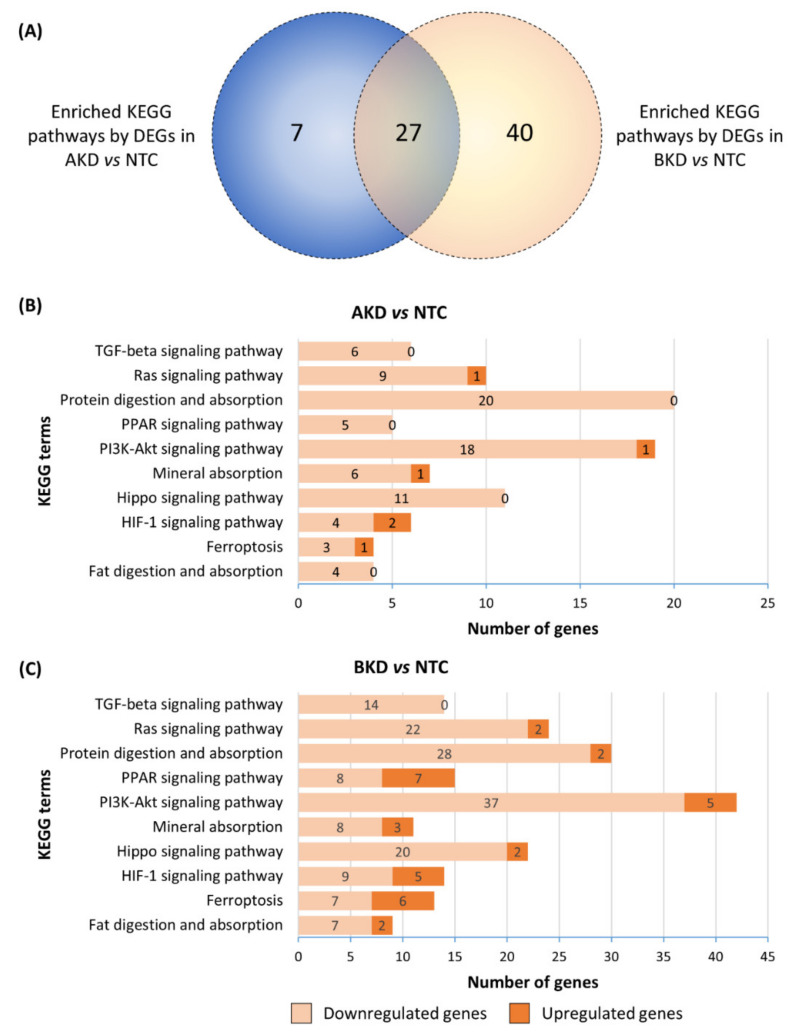
KEGG pathway enrichment analysis of the DEGs in different comparisons. (**A**) Venn plot of the KEGG pathways significantly enriched by the DEGs in AKD vs. NTC and BKD vs. NTC. The figure also shows the top 10 KEGG pathways related to placental or fetal development significantly enriched by the DEGs in (**B**) AKD vs. NTC and (**C**) BKD vs. NTC. A complete list of the KEGG pathways significantly enriched by the DEGs in AKD and BKD TE is provided in Tables S2 and S3.

KEGG pathway enrichment analysis of the DEGs in AKD and BKD TE compared to NTC was performed, and the KEGG pathways with *p* ≤ 0.05 were considered significantly enriched. The DEGs in AKD TE significantly enriched 43 KEGG pathways and the DEGs in BKD TE significantly enriched 67 KEGG pathways, whereas 27 enriched KEGG pathways were common between AKD and BKD TE (Figure 4A). Some of the KEGG pathways related to placental and fetal development, enriched in both AKD and BKD TE, included transforming growth factor-beta signaling pathway, Ras signaling pathway, protein digestion and absorption, peroxisome proliferator-activated receptor (PPAR) signaling pathway, phosphatidylinositol-3 kinase-AKT serine/threonine kinase 1 (PI3K-AKT) signaling pathway, mineral absorption, Hippo signaling pathway, hypoxia-inducible factor 1 (HIF-1) signaling pathway, ferroptosis, and fat digestion and absorption (Figure 4B,C). Although these pathways were enriched in both AKD and BKD TE, the number of DEGs in each enriched KEGG pathway was higher in BKD TE compared to AKD TE (Figure 4B,C).

**Figure 5:**
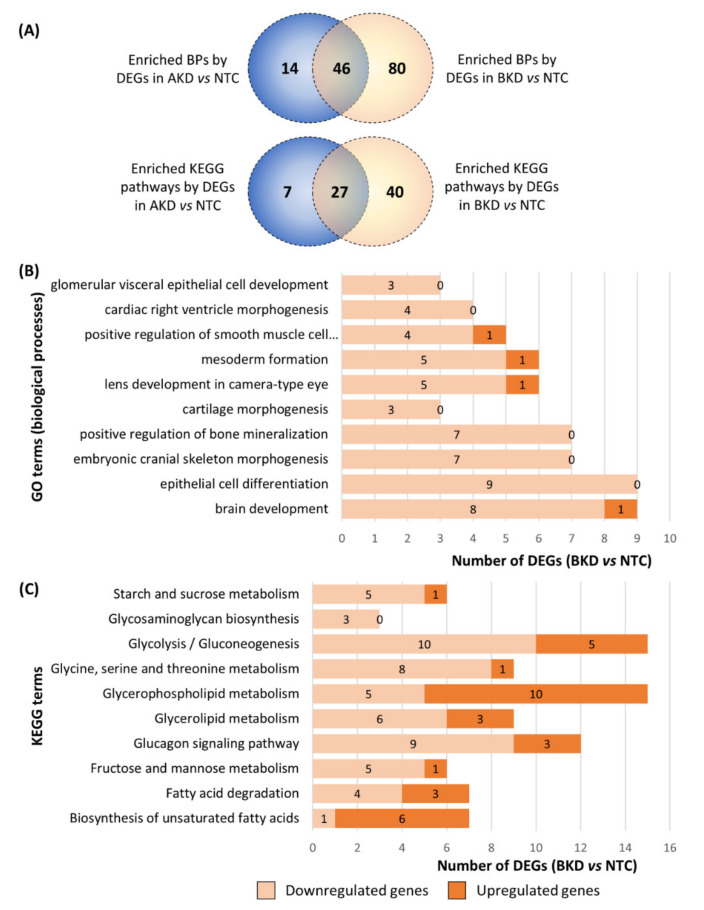
BPs and KEGG pathway enriched only in BKD TE. (**A**) Venn plot of BPs and KEGG pathways significantly enriched by The DEGs in AKD and BKD TE. 80 BPs and 40 KEGG pathways were enriched only in BKD TE. The figure also shows (**B**) 10 BPs related to fetal development and (**C**) 10 KEGG pathways related to metabolism enriched only in BKD TE. A complete list of KEGG pathways significantly enriched by the DEGs in AKD and BKD TE is provided in Tables S2 and S3.

A comparison of the enriched BPs and KEGG pathways in AKD and BKD TE showed that 80 BPs and 40 KEGG pathways were enriched only by the DEGs in BKD TE (Figure 5A). Interestingly, several enriched BPs in BKD TE were related to fetal development, such as glomerular visceral epithelial cell development, cardiac right ventricle morphogenesis, positive regulation of smooth muscle cell proliferation, mesoderm formation, lens development in the camera-type eye, cartilage morphogenesis, positive regulation of bone mineralization, embryonic cranial skeleton morphogenesis, epithelial cell proliferation, and brain development (Figure 5B). All of these BPs were enriched mainly by downregulated genes in BKD TE (Figure 5B). Moreover, several enriched KEGG pathways in BKD TE were related to metabolism, such as starch and sucrose metabolism, glycosaminoglycan biosynthesis, glycolysis/gluconeogenesis, glycine, serine and threonine metabolism, glycerophospholipid metabolism, glycerolipid metabolism, glucagon signaling pathway, fructose and mannose metabolism, fatty acid degradation, and biosynthesis of unsaturated fatty acids (Figure 5C). These KEGG pathways were enriched mainly by downregulated genes, except glycerophospholipid metabolism and biosynthesis of unsaturated fatty acids, which were enriched mainly by upregulated genes in BKD TE (Figure 5C). Gene set enrichment analysis (GSEA) of the DEGs in AKD and BKD TE showed that the hallmark pathway epithelial-mesenchymal transition, and the reactome pathways extracellular matrix organization and collagen formation were significantly enriched in both AKD and BKD TE. A complete list of all enriched hallmark and reactome pathways is provided in Tables S2 and S3.

### 3.3. The Common DEGs in All Comparisons

**Figure 6:**
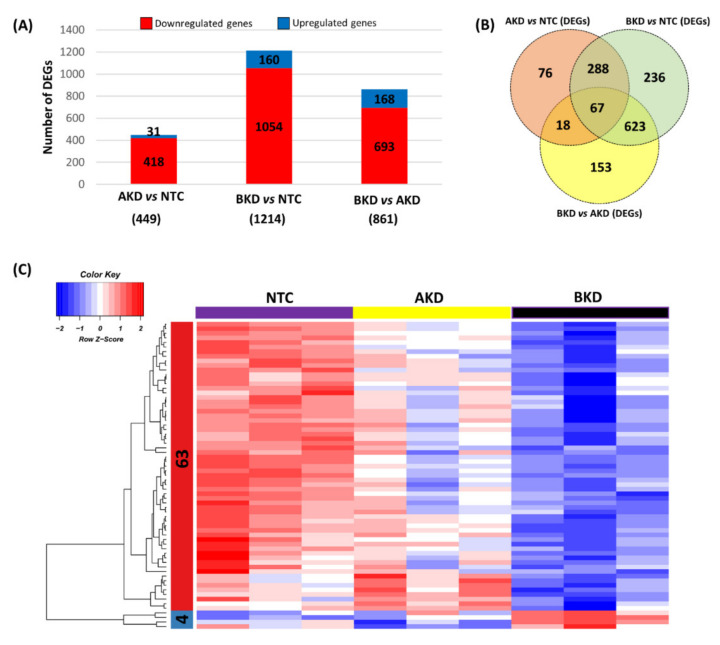
The common DEGs in different comparisons. (**A**) Bar plot showing the upregulated and downregulated DEGs in AKD vs. NTC, BKD vs. NTC, and BKD vs. AKD. (**B**) Venn plot using the total DEGs in AKD vs. NTC, BKD vs. NTC, and BKD vs. AKD. **(C)** The heatmap of the common DEGs in all comparisons. A total of 47 common DEGs were distributed in two clusters based on their co-expression with cut-off criteria of FPKM > 5, |logFC > 2|. In the color key, the red color represents upregulation, the blue color represents downregulation and the white color represents no change in expression.

We found 449 DEGs in AKD vs. NTC (418 downregulated and 31 upregulated), 1214 DEGs in BKD vs. NTC (1054 downregulated and 160 upregulated), and 861 DEGs in BKD vs. AKD (693 downregulated and 168 upregulated) (Figure 6A). Moreover, 67 genes were differentially expressed in all comparisons (Figure 6B). The heatmap, generated using the hierarchical clustering method, showed that 67 common DEGs were grouped in two clusters with 63 genes in cluster 1 and 4 genes in cluster 2 (Figure 6C).

**Figure 7:**
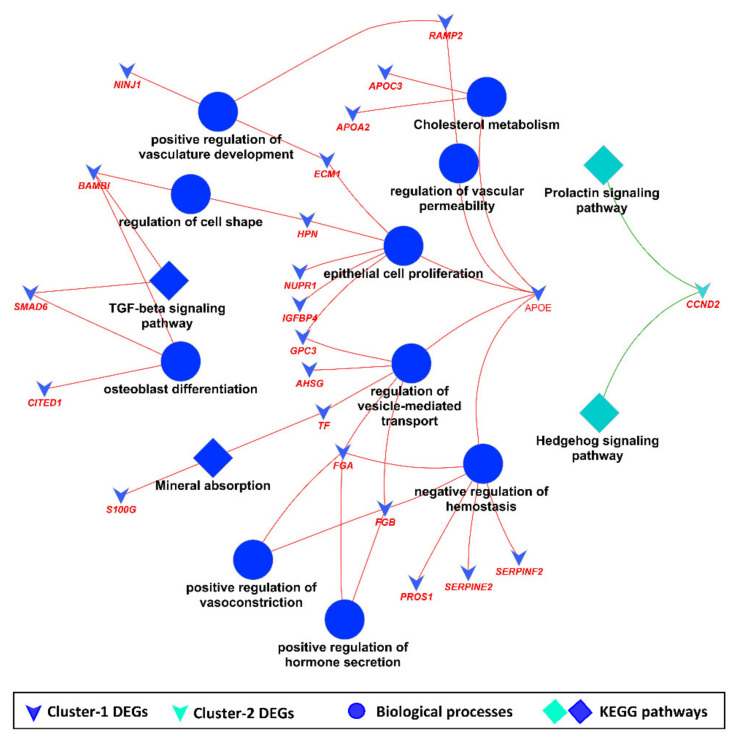
KEGG pathway and GO enrichment analysis of common DEG clusters.

KEGG pathway and GO enrichment analysis of common DEG clusters showed that common DEG clusters enriched important BPs such as cholesterol metabolism, epithelial cell proliferation, positive regulation of vasculature development, and osteoblast differentiation, whereas significantly enriched KEGG pathways included transforming growth factor-beta signaling pathway, mineral absorption, prolactin signaling pathway and the hedgehog signaling pathway (Figure 7).

### 3.4. Let-7 miRNA Target Genes

**Figure 8:**
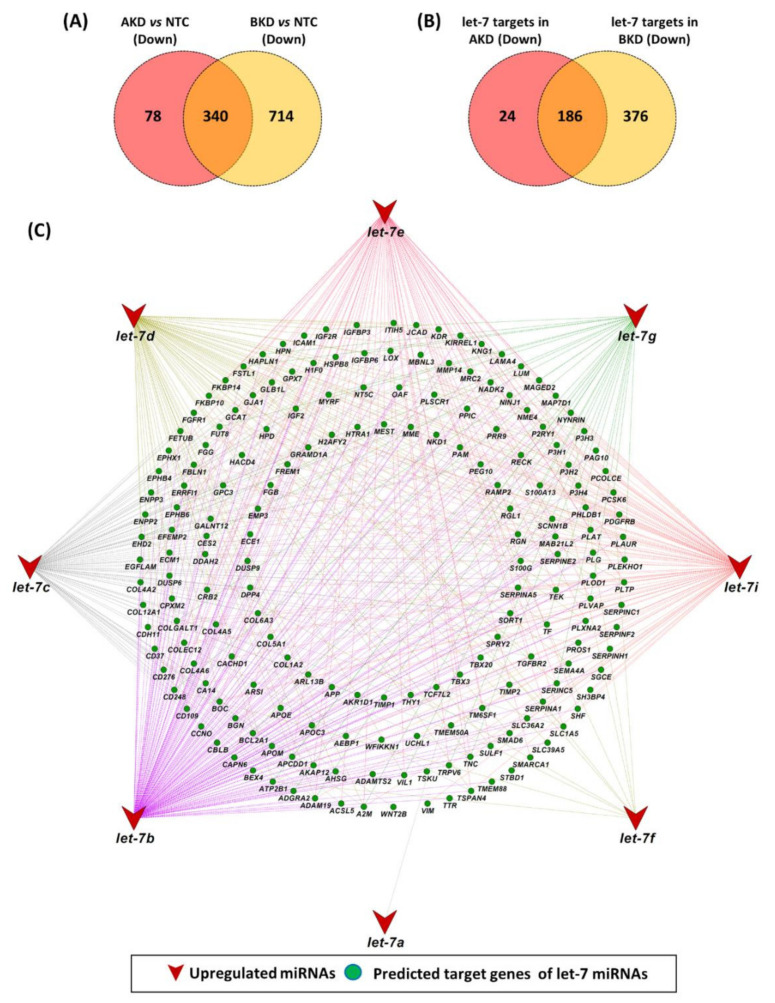
Downregulated target genes of let-7 miRNAs in AKD and BKD TE. Venn plots show (**A**) common downregulated genes and (**B**) common let-7 target genes in AKD and BKD TE. **(C)** miRNA-target gene network showing potential targets of each let-7 miRNA.

We have previously shown that let-7 miRNAs (let-7a, b, c, d, e, f, g, i) were upregulated in AKD and BKD TE compared to NTC [24]. A total of 418 genes were downregulated in AKD vs. NTC and 1054 genes were downregulated in BKD vs. NTC, whereas there were 340 common downregulated genes in both AKD and BKD TE compared to NTC (Figure 8A). Out of 418 downregulated genes in AKD TE, 210 genes were identified as potential targets of let-7 miRNAs (Figure 8B; Table S5). Similarly, out of 1054 downregulated genes in BKD TE, 562 genes were identified as potential targets of let-7 miRNAs (Figure 8B; Table S5). Comparison of downregulated let-7 miRNA target genes showed that 186 let-7 target genes were downregulated in both AKD and BKD TE compared to NTC (Figure 8B). The results further showed that, out of 186 common let-7 target genes, let-7a, let-7b, let-7c, let-7d, let-7e, let-7f, let-7g and let-7i can target 1, 169, 102, 75, 77, 10, 34 and 99 genes respectively (Figure 8C).

**Figure 9:**
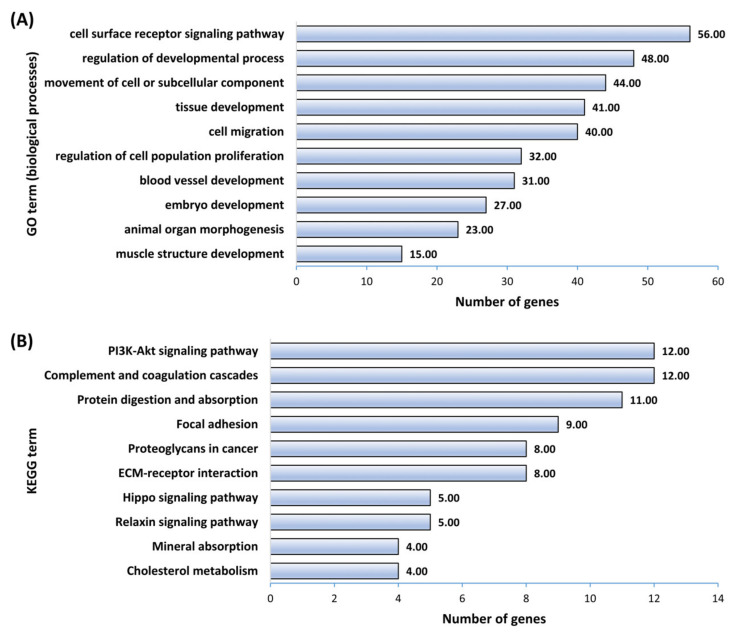
GO and KEGG pathway enrichment analysis for let-7 target genes downregulated in both AKD and BKD TE. Figure shows (**A**) 10 significantly enriched BPs and (**B**) 10 significantly enriched KEGG pathways important for placental or fetal development. A complete list of BPs and KEGG pathways significantly enriched by downregulated let-7 target genes is provided in Table S5.

Enrichment analysis of common let-7 target genes showed that these genes enriched BPs and KEGG pathways important for placental and fetal development. Important BPs significantly enriched by downregulated let-7 target genes included cell surface receptor signaling pathway, regulation of the developmental process, movement of cell or subcellular component, tissue development, cell migration, regulation of cell population proliferation, blood vessel development, embryo development, animal organ morphogenesis, and muscle structure development (Figure 9A). Similarly, important KEGG pathways enriched by downregulated let-7 target genes included PI3K-Akt signaling pathway, protein digestion and absorption, focal adhesion, Hippo signaling pathway, mineral absorption, and cholesterol metabolism (Figure 9B).

### 3.5. Differentially Expressed Transcription Factors

**Figure 10:**
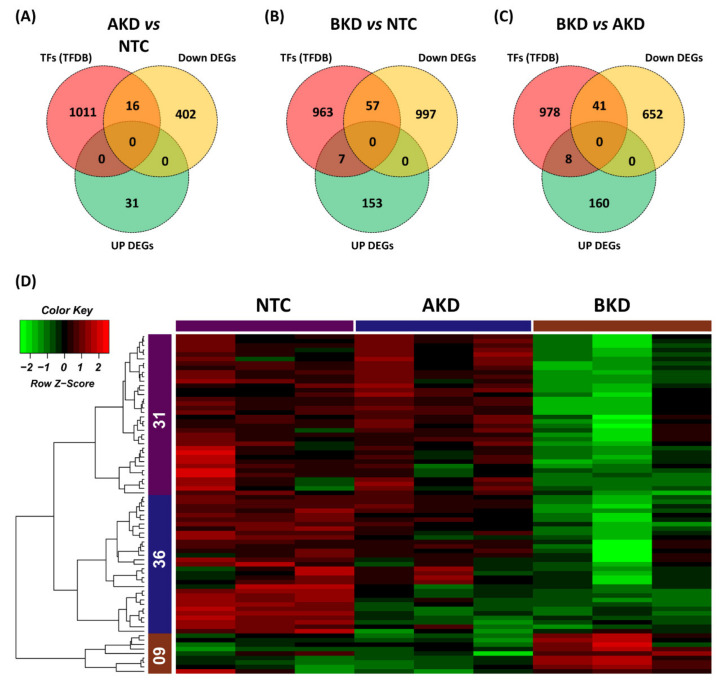
Venn plots to identify differentially expressed transcription factors (TFs) in (**A**) AKD vs. NTC, (**B**) BKD vs. NTC, and (**C**) BKD vs. AKD. (**D**) Heatmap of all differentially expressed TFs. A total of 76 transcription factors were distributed in 3 clusters based on their co-expression with cut-off criteria of FPKM > 5, |logFC > 2|. In the color key, the red color represents upregulation, the green color represents downregulation, and the black color represents no change in expression.

The list of Ovis aries transcription factors (TFs) was obtained from the AnimalTFDB3.0 [32] and Venn plots were used to identify differentially expressed TFs in different comparisons. The analysis showed that 16 TFs were downregulated in AKD vs. NTC (Figure 10A), 57 TFs were downregulated, and 7 TFs were upregulated in BKD vs. NTC (Figure 10B), 41 TFs were downregulated, and 8 TFs were upregulated in BKD vs. AKD (Figure 10C). Heatmap was generated for differentially expressed TFs in all comparisons, and TFs were grouped in 3 clusters with 31 TFs in cluster 1, 36 TFs in cluster 2, and 9 TFs in cluster 3 (Figure 10D). Differentially expressed TFs in all comparisons are listed in Table S6.

**Figure 11:**
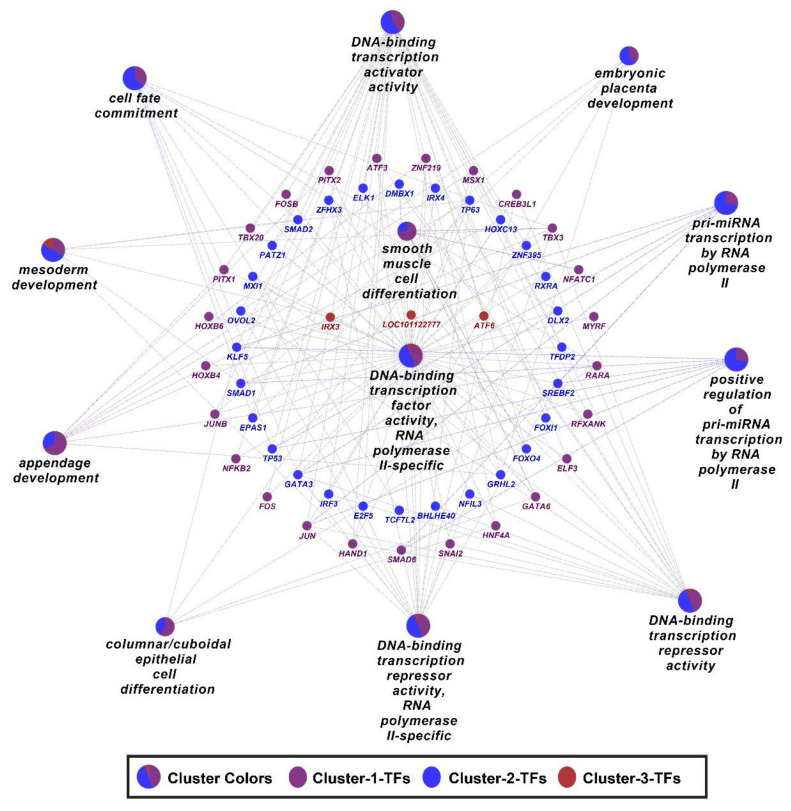
GO enrichment analysis for BPs of 3 clusters of differentially expressed transcription factors. Different pie colors in BPs shapes represent the proportion of TFs from different clusters. A complete list of BPs significantly enriched by differentially expressed TFs is provided in Table S6.

Gene ontology enrichment analysis of differentially expressed TF clusters showed that important BPs such as appendage development and smooth muscle cell differentiation were enriched primarily by TFs in cluster 1, and cell fate commitment, pri-miRNA transcription by RNA polymerase II, embryonic placenta development, and DNA-binding transcription activator activity were enriched primarily by TFs in cluster 2 (Figure 11).

## 4. Discussion

Before its attachment to the uterine epithelium, the ruminants’ blastocyst undergoes an elongation phase and develops into a filamentous conceptus [4,5,6]. The rapid proliferation of trophoblast cells is required for the conceptus elongation [5], and a reduced conceptus elongation can lead to pregnancy loss [11,12,13]. We have previously shown that the trophectoderm-specific knockdown of LIN28A/B in day 9 ovine blastocysts increased the let-7 miRNAs levels in TE and reduced the conceptus elongation at day 16 of gestation [24]. Here we showed that, by regulating the expression of its downstream target genes, the LIN28-let-7 axis is involved in a wide range of biological processes important for placental and fetal development. Compared to NTC, 418 genes were downregulated and 31 genes were upregulated in AKD TE, whereas 1054 genes were downregulated and 160 genes were upregulated in BKD TE. Interestingly, there were 355 common DEGs in AKD vs. NTC and BKD vs. NTC. Despite having many similar functions, including the suppression of let-7 miRNA biogenesis, LIN28A and LIN28B both differ in their subcellular distribution and their mechanisms of suppressing the let-7 miRNAs biogenesis [40,41]. Although the regulation of gene expression by LIN28 through let-7 miRNAs is their most studied aspect, LIN28A/B proteins can also directly bind the mRNAs of several genes and facilitate their translation [42]. According to Hafner et al, LIN28A binds 1803 mRNAs whereas LIN28B binds 4382 mRNAs in human embryonic kidney (HEK293) cells, indicating a greater involvement of LIN28B in gene regulation and cellular processes [42]. They also reported 1674 mRNAs that can bind to both LIN28A and LIN28B proteins [42]. These findings also explain the higher number of DEGs in BKD than in AKD TE, and 355 common DEGs in AKD and BKD TE in our study.

The DEGs in AKD TE significantly enriched 60 BPs and the DEGs in BKD TE significantly enriched 126 BPs. Moreover, 46 identical BPs were enriched in both AKD and BKD TE, including the BPs important for placental development. Collagen is one of the most prevalent compounds at the feto-maternal interface and is thought to play a regulatory role in the trophoblast invasion and migration, induction of immune tolerance at the feto-maternal interface, and angiogenesis [43]. According to our data, genes encoding collagen type I alpha 1 chain (COL1A1), COL1A2, COL3A1, COL5A1, and COL5A2 were downregulated in both AKD and BKD TE. De novo vasculogenesis and angiogenesis are critical for normal placental development and function as well as fetal development [44,45]. In the ruminant placenta, the fetal cotyledons have numerous small capillaries that are highly branched compared to caruncular vasculature [45]. According to our results, the downregulated genes in both AKD and BKD TE significantly enriched the BPs critical for the development of placental vascular bed, including positive regulation of angiogenesis, sprouting angiogenesis, and vasculogenesis. Receptor activity modifying protein 2 (RAMP2) is an essential factor for vascular integrity and angiogenesis [46] and was downregulated in both AKD and BKD TE. The migration of epithelial cells and their interaction with the extracellular matrix is critical during vasculogenesis and angiogenesis [47,48]. Our analysis showed that the genes involved in the positive regulation of epithelial cell migration were downregulated in both AKD and BKD TE.

The extracellular signal-regulated kinase (ERK) cascade is a chain of proteins involved in the important cellular processes, including cell proliferation, survival, differentiation, and apoptosis [49]. ERK1 and ERK2 have been long known for their roles in early embryonic development and different pathological conditions in adult animals [50,51]. Other than its profound role in embryogenesis, appropriate ERK signaling is required for normal placental development [52,53]. According to our data, 9 downregulated genes in AKD TE and 16 downregulated genes in BKD TE were involved in the positive regulation of the ERK1/2 cascade. Similarly, the Wnt signaling pathway plays an important role during the early stages of pregnancy, including blastocyst formation, blastocyst activation, and trophoblast proliferation, invasion, and migration [54]. Our results showed that 5 downregulated genes in AKD TE and 9 downregulated in BKD TE were involved in the positive regulation of the canonical Wnt signaling pathway. In sum, after LIN28A/B KD in TE, dysregulation of the genes regulating the important biological process can lead to severe pregnancy complications.

The DEGs in AKD and BKD TE significantly enriched 43 and 67 KEGG pathways, respectively. There were 27 common KEGG pathways significantly enriched by the DEGs in both AKD and BKD TE, including the pathways important for placental development. Transforming growth factor-beta (TGF-beta) promotes the proliferation and invasion of trophoblast cells, an important phenomenon during early placental development [55,56]. The disruption of TGF-beta signaling negatively impacts trophoblast function and contributes to the pathogenesis of pregnancy-associated disorders [57]. We found that the TGF-beta signaling pathway was enriched by 6 and 14 downregulated genes in AKD and BKD TE, respectively. TGF-beta type 2 receptor (TGFBR2), the key regulator of TGF-beta signaling, was downregulated in both AKD and BKD TE. Peroxisome proliferator-activated receptor (PPAR) signaling regulates the important trophoblast functions such as immune tolerance, invasion, syncytialization, and metabolism [58,59]. According to our results, 5 downregulated genes in AKD TE, and 8 downregulated and 7 upregulated genes in BKD TE are involved in PPAR signaling pathways.

The phosphatidylinositol-3 kinase-AKT serine/threonine kinase 1 (PI3K-AKT) signaling pathway is one of the biggest networks for intracellular signal transduction [60] and regulates trophoblast proliferation, invasion, and migration by its interaction with other signaling pathways [61,62]. Interestingly, the results showed that 18 downregulated genes in AKD TE and 37 downregulated genes in BKD TE were involved in the PI3K-AKT signaling. Similarly, hypoxia-inducible factor 1 (HIF-1) is considered a major regulator of trophoblast function [63], and its aberrant expression in the trophoblast cells can lead to intrauterine growth restriction [64]. The genes involved in the HIF-1 signaling pathway were downregulated in both AKD and BKD TE. The Hippo signaling pathway is a key regulator of organ size and plays a vital role in angiogenesis [65] and the self-renewal of trophoblast progenitor cells in the human placenta [66]. In this study, the Hippo signaling pathway was enriched by 11 and 20 downregulated genes in AKD and BKD TE, respectively. The transfer of nutrients from the mother to the fetus is one of the major functions of the placenta accomplished by different membrane transporters [67]. The placenta can either directly transfer the nutrients from the mother to the fetus or first metabolizes them to alternate forms [68]. According to our results, multiple genes involved in protein digestion and absorption, fat digestion and absorption, and mineral absorption were downregulated in both AKD and BKD TE. We suggest that the dysregulation of the pathways involved in the transplacental transport can lead to nutrient insufficiency in the fetus and affect fetal growth.

Previous studies have shown that the miRNAs or mRNAs of placental origin can be exported to both fetal and maternal compartments [69,70,71,72,73]. In a recent study, we reviewed how the miRNAs of placental origin can be trafficked to the mother and the fetus and affect important biological processes by targeting different genes [22]. Upregulated levels of let-7 miRNAs both in the placenta as well as maternal circulation have been associated with the pathogenesis of pregnancy-associated disorders such as preeclampsia and intrauterine growth restriction [74,75,76,77]. Both AKD and BKD TE reported in this current study had a higher level of let-7 miRNAs compared to NTC [24] and we, based on the previous reports, suggest that these miRNAs can be transferred to both fetal and maternal circulation. Hence, along with affecting the placental development and function, aberrant levels of miRNAs or mRNAs in AKD and BKD TE can also affect fetal development and growth as well as maternal health. 

LIN28A and LIN28B regulate the let-7 miRNA processing by distinct mechanisms and are also thought to regulate different sets of let-7 miRNAs [78]. We have previously reported that let-7 miRNAs were upregulated in the term human placentas from IUGR pregnancies, and LIN28 knockout in human trophoblast cells led to upregulated let-7 miRNAs and reduced cell proliferation [23]. LIN28A knockout upregulated let-7a, let-7b, let-7c, let-7d, and let-7e, whereas LIN28B knockout led to upregulation of let-7a, let-7b, let-7c, let-7d, let-7e, and let-7i in the human trophoblast cells [23]. Moreover, LIN28B knockout had a more rigorous effect on the levels of let-7 miRNAs compared to LIN28A knockout [23]. In this current study, however, all let-7 miRNAs (let-7a, let-7b, let-7c, let-7d, let-7e, let-7f, let-7g, and let-7i) were upregulated in both AKD and BKD TE compared to NTC, and there was no significant difference in the levels of let-7 miRNAs in BKD vs. AKD.

A single miRNA can regulate the expression of multiple target genes [79,80], suggesting their importance in regulating biological processes. The data from this study showed that let-7b can potentially target 169 downregulated genes in both AKD and BKD TE, which reaffirms the concept of multiple target genes of a single miRNA and highlights the importance of let-7b in the regulation of different biological processes. The let-7 target genes enriched the BPs and KEGG pathways important for fetal and placental development. Our analysis further revealed that 76 transcription factors were differentially expressed in all comparisons (AKD vs. NTC, BKD vs. NTC, and BKD vs. AKD). The transcription factors bind to specific motifs in the DNA and act as a major regulator of gene expression [81]. We suggest that, besides the canonical way of regulating the gene expression through let-7 miRNAs, the LIN28-let-7 axis can play a key role in a variety of biological pathways by regulating the expression of transcription factors.

It has been shown in vitro that LIN28 regulates trophoblast proliferation by regulating the proliferation-associated genes. Using an in vivo approach of the trophectoderm-specific gene manipulation here we show that, along with regulating the trophoblast proliferation and conceptus elongation, the LIN28-let-7 axis targets a wide range of genes involved in placental development, fetal development, and metabolism. Based on the existing evidence of the trafficking of the placental miRNAs to both fetal and maternal compartments and the regulation of important biological processes by the let-7 target genes in this current study, we suggest that the elevated levels of let-7 miRNAs in the placenta or maternal circulation can be used to predict or diagnose fetal and placental health. A wide range of the let-7b target genes makes it a strong candidate to become a viable non-invasive biomarker. The reduced conceptus elongation and the dysregulation of important genes suggest that the successful establishment of pregnancy could be challenging subsequent to LIN28 knockdown. Further studies need to be done to explore the use of let-7 miRNAs as the non-invasive biomarkers and the physiological ramifications of LIN28 KD in the establishment and progression of pregnancy.

## Supplementary Materials

The following supporting information can be downloaded at: https://www.mdpi.com/article/10.3390/cells11071234/s1, Table S1: shRNA constructs; Table S2: SC vs. AKD; Table S3: SC vs. BKD; Table S4: AKD vs. BKD; Table S5: let-7 target genes; Table S6: TFs.
